# Penetration of COVID-19 Related Terminology into Legal, Medical, and Journalistic Discourses

**DOI:** 10.1007/s11196-021-09881-3

**Published:** 2022-01-11

**Authors:** Paula Trzaskawka, Joanna Kic-Drgas

**Affiliations:** Adam Mickiewicz University, Poznań, Poland

**Keywords:** COVID pandemic, Specialisation of language, Legal acts, Linguistic changes, Legal lect, Medical lect, Journalistic lect, Student’s t-test

## Abstract

March 2020 has become a moment of change in communication mode and quality. Previously, the media paid attention to the current affairs, however, never earlier the journalistic discourse has been so influentially affected by the ongoing phenomenon as in the case of COVID-19. Almost overnight the new terminological phenomena with specific legal or medical reference were introduced into everyday language mainly via mass media and become an important part of a pandemic related narration. The strong influence on the shape of the mentioned linguistic changes has mainly the adoption of new legal regulations due to the unexpected outbreak of the pandemic. The aim of the following paper is to investigate how COVID-19 pandemic affected the specialisation of the journalistic discourse and how different domains (law, medicine) are being influenced by new terminology and in other way round, how for example law and medicine influence new “COVID language”. In order to take the interdisciplinary nature of the issue into account, the degree of hybridity of the selected texts will be examined by means of selected material analysis. The methodology applied in the paper uses an empirical approach and comparative analysis. The material used for the analysis comes from the selected Polish quality and boulevard press. The paper concerns the linguistic influence of the “invisible enemy” on the language presented in press. The main findings reveal the intense use of neologisms, borrowings, and it shows that the discourse was changed linguistically thanks to Student’s t-test.

## COVID-19 Pandemic Outbreak and Its Influence on Diverse Fields

The unexpected outbreak of COVID-19 pandemic not only paralysed the functioning of basic state institutions but also greatly affected the lives of individuals worldwide. Apart from that the danger of uncontrolled spread of the disease for which there is currently no effective cure has led to a complete change in the approach to medicine. Also, the prevailing pandemic has revealed weaknesses in the health system in most countries. In addition, it is worth mentioning the sudden change in the education system from traditional face-to-face education to digital solutions. The pandemic crisis acted as a catalyst for communication development. Companies, universities, and government departments that have so far been reluctant to adopt new communication technologies have been forced to try out new digital formats. Where experience with them proves positive, the changes introduced will remain. Remote working from home, videoconferencing and e-learning will therefore be increasingly used, especially as appropriate offers in this area will save costs and make employers more attractive.

All processes mentioned above are strictly connected with language. Language is directly linked to highly specialised professional life. “Language plays a prominent role in the communication society. So if business is to evolve and grow, it has to deal with communication. If companies and customers want to find each other, then this can only be done with language” ([26]: 10).

The new reality has forced the governments of many countries to regulate many new phenomena by means of legislation, which has also contributed significantly to the constitution of the new reality. The pace of new waves pushed governments all around the world to act quickly and in haste. Some regulations introduced by them were even in conflict with national constitutions. This contributed to many national discords between citizens, governments and even amongst families. New experiences and objects need new forms of expression that allow them to find their way in the surrounding reality; this is precisely the motivation for developing new (technical) terms or even new quality in modern communication.

Not only specific terms used for description of medical and legal aspects of life appear frequently in COVID-19 related discourse but also the visual aspect of colour zones can be treated as a new semiotic sign for its viewers. Like symbols—all colours represent something and their meaning can differ drastically from one culture to another. Colours can be used as a way of signifying value, emotions, orders and so on. In case of pandemic press narration, it appears that all green, red and yellow/orange areas are universal all around the world.

According to de Saussurean semiology, colour semantics means “[t]he means by which languages communicate the types of visual impression” ([4]: 9). However, colour semantics has a broader meaning. According to Kauppinen-Räisänen and Jauffret the basic premise of colour semantics is that “colour is any sign, verbal or visual, that signifies something other than itself” ([22]: 10). Caivano [5] stresses that “a visual colour possesses the ability to substitute for different things. As a result, colour semantics constitutes a platform to understand and explain colour meanings and relations with the object or context as a form of not only linguistics but also of visual communication” (in Kauppinen-Räisänen and Jauffret). As red, green, yellow or orange can have different associations in different colours it is generally accepted that in case of COVID-19 the green area means an area where people can freely move, the yellow one is the one where people can move but with some restrictions and the red area people are prohibited with movement. It can be compared with traffic lights: green means go, yellow means be careful, you can go but there are some conditions to be fulfilled and the red one which means stop.

Concerning the above-mentioned socio-technological and semiotic changes in language the aim of this article is to analyse how COVID-19 affected the use of specific legally and medically rooted terminology in general press discourse and how they influence each other. The lack of clear and verified information on the pandemic, as well as different medical positions, causes misinformation. Conflicting messages appear in the press, the understanding of which is further complicated by the use of (not always correct) specialist medical or legal terminology.

## Languages for Specific Purposes Versus General Languages

In order to analyse terminology of COVID we have to bear in mind that it appears in different domains and these domains are affecting one another. COVID is of course a disease which can be found in a medical discourse (but under a different name—SARS-CoV-2), then it can be found in legal texts as it was necessary to introduce new laws in countries all around the world to deal with the new type of virus. New implemented laws required introducing new terms to legal discourse. And lastly, the press which is informing wider audience about new regulations or new medical things in simpler way, not specialist. That is why firstly, we have to resort to languages for specific purposes, to discuss what are these, and why exactly the authors have chosen these three particular lects (legal, medical, and journalistic) to discuss the language of COVID. The authors understand lect as a variety of speech having a sociolinguistic or functional identity within a particular speech community (in this case lawyers and judges, doctors, and journalists).

According to Hoffmann ([18]: 53), language is not an isolated creation, but a system of:

linguistic means[s] used in a field of communication that can be limited by subject matter in order to ensure communication between people working in this field (and the popularization of subject matter content as well as contact with certain non-specialists) ([18]: 53).^1^

Languages for specific purposes should not be analysed as separate and independent systems because their inherent structures (grammatical, phonetic, etc.) are identical to the structures of general language.

Fluck ([13]: 16) emphasizes in his remarks that specialised languages are used as means of communication among specialists and that they should therefore be distinguished from common language because of their pragmatic use. Thus, technical languages make use of various elements of the common language and it is worth highlighting that a specialised text is not exclusively composed of specialised words. Moreover, the common language represents a “reservoir” for the formation of new terms.

According to Kalverkämper ([21]: 112ff.), the relationship between specialised and general language is gradable and can be represented on the scale of specificity between “(extremely) feature-rich” and “(extremely) feature-poor” ([28]: 31). However, it is difficult to ascribe concrete linguistic features to this scale of specialized linguistics and thus to elaborate the hierarchy of specialized texts, as pointed out by Hoffmann (1998: 164f.), among others. Therefore, Franciszek Grucza ([16]: 19f.) rightly emphasizes that the boundaries between general language and specialized languages are fluid and dynamic. The primary features by which the distinction between them runs are terminology, textemics (particular types of texts with a higher degree of technicality), and function (cf. [17]: 151).

The reciprocal relationship between general and specialized language has been illustrated by different models based on a vertical or horizontal division of languages [3]. Vertically, language is divided into different levels of language, whereas a horizontal division illustrates the numerous areas of research interest and use within the language (e.g. in business, technology, medicine, law), with further gradations occurring within these levels over time ([2]: 16).

According to Efing [10], general language and specialised languages represent a continuum in which the register terms described in the relevant literature are located, with general language as a comparative and delimiting foil in both professional and technical language. The left–right arrangement defines the proximity or distance from the two poles, while the transverse direction denotes the distinction between orality and writing ([10]: 420). With the help of the triangle, Efing shows the coexistence between the language registers and the blurred boundaries between them.

Delisle (1999: 181) describes language for specific purposes as: “the language used in a given subject field, which comprises both the terminology of the field and the means of expression specific to the field.” The text can be researched in three ways:

1. the language in which it was created,
2. information it contains,
3. the language and paralanguage conventions which decide on its semantic meaning structure or language form.

The influence of specialised languages on the lexis and syntax of the common language leads to the “scientification” of the latter, whereby Maghetiu ([25]: 69) argues that it is not a question of the vocabulary itself, but rather of the dissemination of scientific-technical thinking, an intellectualisation of the language, which refers above all to the possibility of expressing abstract facts, as well as a tendency towards precision, information density and economy of expression.

In the lexical field, the intellectualisation of the common language takes place through:

1. occurrence of specialised terms for objects and phenomena of intellectual and material culture, which penetrate from the technical languages into the common language;
2. the need for new terms that arises with the development in the field of material culture and is characterised by the specialisation of common language words [13]: 167).

According to ([25]: 70), the progressive specialisation of the common language can result in communication breakdowns and problems of understanding.

In the case of COVID the authors have decided to resort to three languages for specific purposes: legal, medical, and journalistic. Each of this type of discourse is crucial to understand the correlations between them. Therefore, they will be briefly characterized below.

## Legal Language

Law and language have always existed in tandem. Moreover, language has always been carrying out the meaning of law. According to Marcus Galdia ([14]: 63) the language of law includes specialized legal terminology, which is the subject of research and study by lawyers and non-lawyers. However, the use of language in legal texts varies considerably. Galdia ([14]: 91) distinguishes a division of the legal language in the following way:

1. language of normative acts (language of legislation),
2. language of legal decisions containing descriptions of facts,
3. language of doctrine,
4. language used by lawyers in professional discussions and in court,
5. language used by laymen in the legal context (last wills and testaments, comments on legal provisions),
6. language used by officials.

On the other hand, Jean-Claude Gémar (after [30]: 17) distinguished three types of legal texts. The first category includes texts such as laws, regulations, judgments, and international agreements. The second category includes: contracts, agreements, forms, last wills and testaments etc. Scientific texts on law belong to the third and smallest category of legal texts.

From the translative point of view, the division of the legal language proposed by Zieliński [31] seems to be the most exhaustive. However, it should be noted that the language of law is not regarded as an autonomous language, but rather as a functional and situational variant of the official language or a style between scientific and official (cf. Wojtak after [20]: 20). According to Franciszek Grucza [15], legal language is the so-called technolect, or a specialised language. Grucza emphasizes that legal language is closely related to a given language, nation, and culture. This makes it different from others, sometimes untranslatable, which is mainly due to the differences that occur in countries with different political and legal systems. Therefore, the most precise statement seems to be that the legal language is a specialized language that has a range of specialized vocabulary, which is more difficult (compared to other fields) and less understandable due to their complexity and specificity, primarily for a layman. As a result, this means that language is both embedded in and manifested in culture. Therefore, translation is an attempt to recreate the meaning of one culture with the help of another language (cf. Nobles and Schiff [27]: 114).

All in all, legal texts are always texts of some specific legislative acts (laws, regulations, orders or resolutions). Legal texts and their language are distinct from other specialized languages and have a different degree of difficulty compared to texts in other fields.

## Medical Language

Medical language because of its specifity belongs to the group of Languages for Specific Purposes reflecting characteristic linguistic and stylistic features. Medical texts are specific, use specialised terminology (the usage of different features—hedging (to avoid making a clear, direct response or statement in the diagnosis), eponyms [in this case these are “terms adapted from the names of famous physicians or scientists” ([19]: 57)], abbreviations or acronyms) and are characterised by complexity and precision. They require the translation of various documents. What should be stressed is the fact that medical translation is claimed to be “the most universal and oldest form of scientific translation because of ubiquitousness of human anatomy and physiology (after all, the human body is much the same everywhere), the long, venerable and well-documented history of medicine, and the hitherto uniform character of the language of medicine, at least in the West” ([12]: 1–2).

The purpose of Language for Specific Purposes (LSP) is to convey objective information that reflects the actual state of the described phenomenon. Medical language is characterised by a neutral, impersonal, and homogenous style (cf. [24], 29.

There are different types of medical texts. According to Kościałkowska-Okońska ([24]: 189–190) and Strębska-Liszewska ([29]: 40), four basic types of medical texts can be distinguished as follows:

- regulatory documents (all documents required by authorities or ethics committees to authorise the marketing of medicinal products and devices);
- educational and informative texts, which are addressed to specific target audiences (e.g. physicians, medical students, patients);
- medical records regarding the patient’s health condition (e.g. results of diagnostic tests, vaccination cards);
- manuals (e.g. for medical equipment or diagnostic equipment).

How medicine and law are linked together? Medical translation is necessary for both legal and market-related reasons. According to Kościałkowska-Okońska ([23]: 8).

legal requirements cover all commercialised medicines with the approval of the Federal Drug Agency or the European Medicines Agency. Additionally, as Poland is one of EU member states, the entire legislation must be translated into Polish. This includes all EU directives: medicine and related fields are concerned mostly with the Medical Device Directive, In Vitro Diagnostics Directive, Active Implantable Device Directive, or Human Medicines Directive.

In times of COVID-19 pandemic it should be stressed that both language of medicine and legal language are related. Medically specific terminology has to be incorporated to legal acts, regulations in terms to properly interpret the same phenomena. In some cases, it will be visible that terminology concerning COVID will belong to both: legal and medical lect. The last lect, which mostly interpret the new medical discoveries or new legal acts or regulations is the journalistic lect. However, in press we will find all lects—legal and medical, as well as journalistic—in cases when legal and medical lect is too difficult to understand or is hard to quickly comprehend by a layman.

## Journalistic Language

Journalistic or publicist style is specific to mass media because it aims at a heterogeneous audience, with a different professional training or various levels of culture. It includes a wide variety of texts commenting on topics of social, economic, and political character, explained for a general comprehension (cf. [11]. This leads on the one hand to the broad diversity of journalistic style and, on the other hand to the complex use specific terminology. Sometimes one term characterises two separate phenomena typical of a particular medium (cf. [8]).

Fer [11] highlights the following features of journalistic language:

1. Using linguistic clichés such as Bucharest—the little Paris or Russia—the Mother country;
2. Calls to processes designed to capture reader’s attention such as: exciting titles and subtitles, usage of pictures and graphics (the colour);
3. Accuracy—is style’ ability to use words strictly necessary to communication, to find the words to perfectly express the idea that the speaker wishes to transmit. Deviations: tiring crowding of words and digression (deviation from the topic);
4. Purity—is given by the strict usage of the words admitted by the cultivated style of language. Deviations arise from ignorance, lack of reading, unconscious imitation and abusive use of neologisms and barbarisms;
5. Naturalness—a clear expression with a connection;
6. Simplicity—is obtained by the use of accessible words, common terms, an ordinary everyday style and by choosing the word that best expresses the idea.
7. The journalistic style characteristics are determined by the aim of press texts, which are written to attract reader’s attention, to inform about the latest issues (cf. [11]. The mentioned functions reflect the extensive use of neologisms and specific terminology for naming new objects or phenomena usually coming from different specific domains.

The lexis of the journalistic variety of language discussed is enriched with neologisms, both word-formative and semantic. In addition to the neutral vocabulary, there are also the words affectionate characterisation. There is a visible mannerism consisting in a kind of worship for non-native words (mainly of English origin). The degree of development of the used vocabulary scope depends on, for example, the scope of a given magazine range, for example local newspapers do not have an extensive range of vocabulary (cf. [8]).

The analysis of press texts shows that in journalistic style there is a class of texts which composition can be presented as a set of three interactions:

1. evaluative (aimed at assessing the conflict relation);
2. question–answer interaction (aimed at looking for or explaining to the reader the causes of the conflict and identifying its perpetrators);
3. motivating (proposing a way of removing conflict) (cf. [7]).

Each interaction is expressed in its own original set of linguistic means (mainly stylistic and rhetoric figures), shaping the systematic nature of speech in interaction.

The topic of the spreading pandemic has dominated the media for nearly 1.5 years, at the same time significantly modifying the specificity of the message. The specificity of the phenomenon described (disease) favours the use of medical terminology, but at the same time regulations issued by governments to standardise preventive measures are also important.

## Research Methods and Material

The used methods are based on a comparative legal analysis and empirical observation. The comparative legal analysis is the analysis of different laws or legal systems through the use of one or more approaches—in our case—EU regulations versus national regulations. The second method was the analysis of different lects (the authors have distinguished three specialist lects which exist in press in relationship, that is to say: legal lect, medical lect and journalistic lect). The languages for specific purposes were discussed in previous section where the authors resorted to legal, medical, and journalistic language. The third method is based on terminological analysis of the corpus consisting of terminology which appears in Polish press. The last method was based on the empirical analysis. The authors by reading news online and in paper versions of newspaper extracted interesting terms which are new in the face of pandemic or are known and previously used but because of the pandemic its usage and frequency multiplied.

The research material can be divided into two categories. The first one which is bigger are the online texts from different Polish magazines/newspapers/news portals. The second category is based on the paper version of Polish newspapers (in this case only one type of a newspaper—Wyborcza) which is smaller as the authors mostly read the news in the Internet. In the brackets we will find the number of articles for a specific source, number of words and the date of publishing).

Online sources:

1. Rzeczpospolita (16 articles, in total: 7178 words; published between 1–31 March 2020, 1–30 December 2019, 18–31 May 2021);
2. Do Rzeczy (1 article, in total: 249 words; published 31st May 2021);
3. Wyborcza (1 article, in total: 2056 words; published 6th September 2021);
4. msn news (1 article, in total:1904 words; published 4th May 2021);
5. Polsat news (3 articles, in total: 1320 words; published 25th December 2020, 28th March 2021 and in 8th September 2021);
6. Interia (1 article, in total: 243 words; published 29th March 2021);
7. DW (1 article, in total: 459 words; published 13th March 2020);
8. Opowiecie.info (1 article, in total: 541 words; published 22nd March 2020).

Paper sources:

1. Wyborcza (10 articles; published between 10–20 September 2020).

## Research Hypotheses

In the next section the authors would like to discuss more broadly the main features of the COVID-19 pandemic narrative conveyed by the mass media and its influence on the medical and legal lect. These three lects: legal, medical, and journalistic are being mixed in the press and sometimes only term from one lect is present in press. It should be stressed that all these three lects are influencing one another. In order to estimate the mentioned impact, the following research questions were addressed:

RQ1: What features of a COVID language/narrative conveyed by mass media can be identified?

RQ2: Basing on the categorized features can a COVID related terminology be classified as a separate domain?

RQ3: Can COVID terminology be enlisted as a category of LSP languages?

## Data Collection and Analysis

In order to excerpt the interesting passages, the daily newspapers (both in online and paper version) were taken into account. The collected articles have been analysed for specialized vocabulary from the fields of medicine and law, and the most frequently occurring terms have been extracted and checked for the highest frequency of occurrence using advanced Google Search (the frequency of them can be found in the Student’s t-test section). In the Table 1, we will find the extracted terms from the previously discussed newspapers. The authors chose them as the frequency of these terms was quite high in almost every researched source. However, some of them were newly created terms (as in the case of “koronaferie” (En. COVID related school break)) or borrowed from different domain (e.g. “cyfrowy bliźniak” (En, digital twin) taken from IT). The authors divided the terms in two groups: (1) terminology strictly coined during the COVID-19 pandemic, and (2) terminology known and used before the outbreak of COVID-19 pandemic. These two groups of terms were chosen in order to check in what way COVID-19 pandemic enriched Polish language of new terminology and new concepts (the statistics are provided in the Student’s t-test section). The terms that are in the Tables 1 and 2 will be discussed in detail in the next section.

**Table 1:** The list of terminology strictly coined during the COVID-19 pandemic (own elaboration)

Terminology strictly coined during the COVID-19 pandemic	
paszport covidowy (En. COVID passport)	lockdown
Unijny Certyfikat Covid (En. The EU Digital COVID Certificate)	koronakryzys (En. coronacrisis)
certyfikat covidowy (En. COVID certificate)	koronatransport (En. coronatransport)
cyfrowy paszport covidowy (digital COVID passport)	koronafobia (En. coronafobia)
unijne cyfrowe zaświadczenie COVID (En. The EU Digital COVID Certificate)	koronasceptyk (En. coronasceptic)
przepustka covidowa (En. covid pass)	covidiota (En. covidiot)
godziny dla seniorów (En. senior citizens’ hours)	wariant brytyjski (En. British variant)
koronaferie (En. COVID related school break)	wariant południowoafrykański (En. South-African variant)
	wariant indyjski (En. Indian variant)

*Data collected as of 29–30.09.2021*

**Table 2:** The list of terminology known and used before the outbreak or COVID-19 pandemic (own elaboration)

Terminology known and used before the outbreak or COVID-19 pandemic	
teleporada (En. phone doctor’s consultation)	pandemia (En. pandemic)
cyfrowy bliźniak (En. digital twin)	epidemia (En. epidemic)
NOP (En. VAE)	namordnik (En. muzzle)
niepożądany odczyn poszczepienny (En. vaccine adverse event)	

*Data collected as of 29–30.09.2021*

## Results and Discussion

In this section the authors will discuss COVID specific terminology used in newspapers and boulevard press. Various types of text analysis use different methods and specialized vocabulary. The authors would like to present some of the methods and some of the COVID specific terms in this paper. Each term has specific features which will be discussed further in detail. The terminology discussed is characterized with:

- borrowings (e.g. internationalisms),
- abbreviations,
- eponyms,
- toponyms,
- derived words, and
- neologisms.

### The Language of COVID—Terminology

Lawyers have their own language, doctors have their own language. Each specific domain has its own jargon or specialized terminology. Or is it just a mixture of different domains where in everyday life we are using terms that we already know but in different context (as well as giving a new meaning to the terms which are new in the new reality that we are since the outbreak of COVID-19 in 2020)? It appears that COVID terminology is partly based on medical language but in majority cases new terms and collocations have been created as this type of virus was not very well known in previous decades.^2^

Below we would like to discuss some of the Polish terms which are either specific, coined during the COVID-19 pandemic or known and used before the pandemic but now their frequency is much higher.

i. paszport COVIDowy (En. COVID passport).

Already in 2020 some countries in the world started issuing documents which confirm if a person recovered from COVID-19. In February 2021, Israel introduced “green pass” system which allows those who are fully vaccinated (or otherwise have recovered fully from COVID-19) to eat at restaurants, attend concerts, and travel to other nations. At the beginning of 2021 the governments of many European countries started the debate about introducing COVID passports which now is in force under title The EU Digital COVID Certificate (EUDCC), (also called the Green Pass), since 1 July 2021 and it eases travel across all 27 member states, and additionally Switzerland, Iceland, Norway, and Liechtenstein.^3^ In Poland and the EU, the following legal acts regulate the principles of granting and applying the EU Digital COVID Certificates:

1. EU Regulations of the European Parliament and the Council of Europe of June 14, 2021 introducing the legal basis for the cross-border use of the EU Covid Certificate system that is to say:
  - Regulation (EU) 2021/953 of the European Parliament and of the Council of 14 June 2021 on a framework for the issuance, verification and acceptance of interoperable COVID-19 vaccination, test and recovery certificates (EU Digital COVID Certificate) to facilitate free movement during the COVID-19 pandemic;
  - Regulation (EU) 2021/954 of the European Parliament and of the Council of 14 June 2021 on a framework for the issuance, verification and acceptance of interoperable COVID-19 vaccination, test and recovery certificates (EU Digital COVID Certificate) with regard to third-country nationals legally staying or residing in the territories of Member States during the COVID-19 pandemic.

The central IT infrastructure was technically ready from June 1, 2021. From that date, the period of connecting national servers of individual Member States had begun, which ended with the launch of the system in the EU on July 1, 2021 (with a six-week transition period for countries that require more time to introduce these digital solutions). The legal act in Poland regulating the processing of the data of vaccinated persons in order to verify that a given person has the status of a vaccinated person against COVID-19 is the Polish Regulation of the Council of Ministers of May 6, 2021 on establishing certain restrictions, orders and bans in connection with an epidemic (Dz. U. 2021.861 with further amendments).^4^

What is more interesting is the fact that COVID passport has many different names all around the world (for example an *immunity passport* or *vaccine passport* which are known for a very long time but in COVID cases the new names have started to appear—a *vaccination ID*^5^ which was used by media (Chicago Sun Times Editorial Board (CNN) in August 2021 in America); *recovery certificate*^6^ used by Edmond in June 2020 during World Economic Forum; *immunity certificate*^7^ (also Chicago Sun-Times April 2020); *release certificate*^8^ in Indian media (May 2020)), it even happens to have three different names in Polish language, that is to say “paszport covidowy”, “certyfikat covidowy” and “cyfrowy paszport covidowy” which is compound of the two previous terms. Below we will discuss briefly the Polish variants of naming this document.

As Poland is in the EU the regulation that applies uses the term “Unijny Certyfikat Covid” which belongs to legal lect and is present in press. On the website pacjent.gov.pl which is a Polish website devoted to medical matters (but it is a governmental website) we have a mixture of legal and medical lects. However, the term that appears there is also “Unijny Certyfikat Covid”. Nevertheless, there are other terms used in press as well. “Paszport covidowy” or “certyfikat covidowy” attests that its bearer is immune to COVID-19. This document is issued in both forms—paper and digital. These two terms are used in Polish language interchangeably. According to the data in Polish National Corpus (a linguistic corpus with a collection of texts where one can find the typical use of a single word or a phrase, as well as their meaning and grammatical function) it is visible that the frequency of the word “paszport” had its peak in 1970. and it is constantly decreasing. When it comes to the term “certyfikat” the situation is reversed. It is more and more frequent to use the term certificate in Polish comparing with the word passport. However, in terms of Covid both versions are used in the media however in official documentation the term “certyfikat” is used. Moreover, in other media (Polish newspaper *Do Rzeczy*) we can find a very similar term “przepustka covidowa” which is used mostly in the context of Italy. In July there was a huge debate in Italy whether covid pass should be obligatory. 2/3 of Italians do not mind having such pass to enter restaurants and other facilities, however 1/3 claims that this is a violation of freedom. Even though covid pass was introduced in August 2021. As it is regulated in Regulation (EU) 2021/953 of the European Parliament and of the Council of 14 June 2021 the used term there is “unijne cyfrowe zaświadczenie COVID”. It seems that in press we have a mixture of all three lects concerning the so-called “covid passports”.

2. Cyfrowy bliźniak (En. digital twin)

Another very interesting term which is used in artificial intelligence and appeared in media in the context of Covid is “cyfrowy bliźniak” (En. digital twin). A digital twin is a new treatment for the effects of Covid-19. The main idea is to build virtual models identical to the existing ones infected with the coronavirus. These so-called digital twins will help heal the effects of chronic Covid-19 syndrome. Thanks to this technology, supported by artificial intelligence and machine learning, personalized health risk assessments and recommendations for drug therapies based not only on individual medical history, but also on the genetic conditions of individual patients will be created. Digital twins are to support hospitals and research centers all around the world. It was the idea of Dell Technologies together with a non-profit organization i2b2 tranSMART foundation. Dell hopes that the use of innovative technology and scale of operations will contribute to the improvement of health. In the future, they will be able to update digital twins with real-time clinical data collected by ventilators and cardiac monitors. This term appeared in a legal context for example in Commission Communication to the European Parliament, Council, European Economic and Social Committee and the Regions Committee; European data strategy from 2020. To sum up, this term appears in three lects: legal (in the previously discussed EU Communication), in journalistic lect (in press), but also in artificial intelligence lect which is not discussed here. It is visible that also other lects (from different domains) are present in press concerning the topic of COVID.

3. NOP (En. VAE)

The next term under discussion is “niepożądany odczyn poszczepienny” which in the context of COVID appears very often. The term may be found for example in the Polish Regulation of the Minister of Health of 23 December 2002 on a vaccine adverse event. In English we use a term a vaccine adverse event (VAE), sometimes referred to as a vaccine injury. VAE is an adverse event caused by vaccination (cf. Canada, Health [6]). The World Health Organization (WHO) knows VAEs as Adverse Events Following Immunization (AEFI) (WHO 2020). It appears that vaccines are not perfect and have many adverse effects. In terms of Covid vaccines there are different adverse effects which depends on the type of a vaccine or the pharmaceutical company that released them, or even the special part of a particular vaccine which was deadly or highly harmful. In the case of NOP—all three lects are in relationship because in every discussed lect the used term is the same.

4. Pandemia *versus* epidemia (En. pandemic vs. epidemic)

The next set of terms in Polish is “pandemia” versus “epidemia” (En. panedemic vs. epidemic). The term “epidemia” appears for example in the Polish Regulation of the Council of Ministers of August 27, 2021 amending the regulation on the establishment of certain restrictions, orders and bans in connection with an epidemic. Sometimes these two terms “pandemic” and “epidemic” are used interchangeably which is incorrect both in Polish and in English. A pandemic (from Greek πᾶν, pan, “all” and δῆμος, demos, “local people” the ‘crowd’) is an epidemic of an infectious disease that has spread across a large region, for instance multiple continents or worldwide, affecting a substantial number of people. Furthermore, pandemic is a hyponym term for epidemic as epidemic (from Greek ἐπί epi “upon or above” and δῆμος demos “people”) is the rapid spread of disease to a large number of people in a given population within a short period of time. In *English Medical Dictionary* (1987) there are the following definitions of the words epidemic and pandemic: “Epidemic—(infectious disease) which spreads quickly through a large part of the population” whereas “Pandemic—(epidemic disease) which affects many parts of the world”. The World Health Organization defines a pandemic as “an epidemic occurring worldwide, or over a very wide area, crossing international boundaries and usually affecting a large number of people” (definition taken from the WHO website^9^). A pandemic is a type of epidemic (one with greater range and coverage), an outbreak of a disease that occurs over a wide geographic area and affects an exceptionally high proportion of the population. While a pandemic may be characterized as a type of epidemic, you would not say that an epidemic is a type of pandemic. In the case of lects it appears that in legal lect mostly the term epidemic occurs, in medical and journalistic lects both terms “epidemic” and “pandemic” are present.

5. Lockdown

The term “lockdown” is a borrowed term which appears to be also an internationalism. The internationalism or international word is a loanword that occurs in several different languages with the same or at least similar meaning and etymology. Furthermore, pronunciation and orthography are similar so that the word is understandable in different languages. Here, as an example can serve the word *lockdown*. *Lockdown* is a restriction policy for people or community to stay where they are, usually due to specific risks to themselves or to others if they can move and interact freely. This term was used globally after the outbreak of COVID-19 in 2020. The first lockdown implemented as a preventive measure in response to COVID-19 was in Wuhan in January 2020.^10^ Many countries introduced lockdowns or, “stay-at-home” propaganda to counteract the effects of spreading the virus. Lockdowns are used for the specific areas, specific locations or even the whole country to limit movements or activities such that only organizations supplying basic needs and services can function normally.

The international term *lockdown* (other forms in English lock-down and lock down) can be found for example in the following languages:

Polish: *lockdown.*

English: *lockdown.*

Dutch: *lockdown.*

Icelandic: *lokkdán.*

Italian: *lockdown.*

German: *der Lockdown.*

Russian: *лoкдayн*.

The etymology of the term *lockdown* comes from English verb phrase *to lock down* which means to secure; make people stay locked indoors for their safety. The term “lockdown” is present in legal and journalistic lects.

### Expressions for Naming the New Phenomenon

1. Godziny dla seniorów (En. senior citizens’ hours).

In order to make it as comfortable and safe as possible for senior citizens to take care of the essentials of everyday life, such as shopping, hours for senior citizens were introduced at various times during the pandemic. Seniors’ hours were introduced for the first time by means of the Polish Council of Ministers’ Ordinance on the Establishment of Certain Restrictions, Orders and Prohibitions in Connection with the Emergency of 9 October 2020 (and then in Regulation of the Council of Ministers of October 14, 2020 amending the regulation on the establishment of certain restrictions and prohibitions in relation to an epidemic). The solution introduced by the government made it possible for seniors to make purchases on weekdays between the hours of 10:00 and 12:00 without having to interact with other customers. However, at different stages of the pandemic, the group entitled to benefit from this solution was defined slightly differently. In the case of the first lockdown, people aged 65+ were allowed to shop during senior citizen hours. During the second lockdown, on the other hand, people aged 60 and over could use this option. Senior citizen’s hours were available in shops, pharmacies, chemists, and the post office. It did not apply to shopping at petrol stations and wholesalers, as well as in DIY and clothing shops.

The government has introduced such restrictions so that the oldest citizens, who are at the highest risk of contracting COVID-19, can isolate themselves from large gatherings of people. Interestingly, the noun senior is noteworthy. It has effectively displaced terms such as old people, pensioners, or the elderly. To sum up, the term “godziny dla seniorów” is present both in legal and journalistic lect. However, it is not present in the medical lect.

2. Teleporada (En. phone doctor’s consultation).

The term “teleporada” became very popular after the outbreak of COVID-19 pandemic when the majority of public doctor’s offices were closed. Doctors started offering a consultation conducted over the phone or using a video call application. A doctor should be in the office or in such a place where bystanders cannot hear him. This term appears in the Polish Regulation of the Minister of Health of 5 March 2021 amending the regulation on the organizational standard of phone doctor’s consultation in primary health care. The term “teleporada” is present in all discussed lects.

3. Koronaferie (En. COVID related school break).

Students, initially disoriented by the sudden shift to remote learning in March 2020, found that they had COVID-related school break. This increment expresses the chaos in the organization of distance learning at home. Not every student had computer equipment, and teachers were only just implementing the use of communication and learning platforms. The least persistent in overcoming technical problems stated that it was a pandemic holiday. This term is present only in journalistic lect. In this case a neologism was created in order to resort to something old and something new. A neologism is a newly created or borrowed word or term from another language. Moreover, the new meaning of an already existing lexeme in a given language will be also called a neologism [9].

4. Namordnik (En. muzzle)

The term “namordnik” is a borrowed term from Russian for a mask or half-helmet which means ‘muzzle’. It originated from a not very elegant prepositional phrase for muzzle. The association with a muzzle suggests the enslavement of society. A borrowing is a translation technique in which in our target text we do not change the original word or phrase which belongs to the initial text, because the target language does not have a proper equivalent [9]. The term “namordnik” does not function in legal or medical lect. In legal and medical lect the term “maseczka” or “maska” are widely used. The term “namordink” in this particular case is a colloquialism which was present in journalistic lect.

5. Koronakryzys, koronatransport, koronafobia (En. coronacrisis, coronatransport, coronafobia).

New words after the outbreak of COVID-19 pandemic appeared. The very frequent use is the *corona* as the abbreviation (abbreviation is taken from the Latin word *brevis* which is a shortened form of a word or phrase; usually, but not always, it consists of a letter or a group of letters taken from the word or phrase). Corona—the first component of compound words indicating their meaning in relation to the pandemic SARS-CoV-2. The abbreviation *corona* has become very productive in creation of new words like: coronacrisis, coronatransport, coronafobia. These terms are used in journalistic lect, sometimes we can hear in media politics using these words, but they do not appear in legal written lect.

6. Koronasceptyk, covidiota (En. coronasceptic, covidiot).

Latin and Greek have contributed thousands of words that have become integrated into many languages’ vocabulary. Derived words are simply borrowings from Latin and Greek. For example, *coronasceptic* and *covidiot* are both derivatives of Latin words and refer respectively to those who accept without much criticism the covidium regulations imposed and those who doubt them. These terms are used in a journalistic lect.

7. Wariant brytyjski, wariant południowoafrykański, wariant indyjski (En. British variant, South-African variant, Indian variant).

In terms of COVID-19 terminology we can find different types of toponyms (toponymy is the study of place names [toponyms], their origins, meanings, use and typology) concerning places where the biggest outburst of the virus appeared. For example, in Poland we can distinguish the following examples:

1. British,
2. South-African,
3. Indian.

Moreover, some of these names after places are called with the Greek alphabet letter (different variants of virus—delta, lambda, etc.). Another example depicting the scale of vaccinations in Poland from *wyborcza.pl*: “Szczepimy się od Giewontu po Pustynię Błędowską” (En. We vaccinate from Giewont to Pustynia Błędowska). Giewont is the name of a peak in the mountain range in southern Poland, while Pustynia Błedowska is a desert located in southern Poland. The other used toponym is Oxford in *medonet.pl*: “Oksfordzka szczepionka na koronawirusa. Co o niej wiemy?” (En. Oxford’s coronavirus vaccine. What do we know about it?” Use of the name of the place where the substance is produced instead of using its proper name (Astra Zeneca) in reference to the vaccination. Moreover, we have extracted one example of an eponym (a name of a person, whether real or fictitious, after which a particular place, tribe, era, discovery, or other item is named or thought to be named) from *opowiecie.info*: “Koronawirus. Czy doświadczymy przepowiedni królowej z Saby?” (En. Coronavirus. Will we experience the prophecy of the Queen of Sheba?). This headline used a reference to the Queen of Sheba, who could foretell the future. These terms are used in journalistic lect, sometimes it is used by politics but only in a spoken legal lect.

## Student’s t-Test

According to Britannica^11^ Student’s t-test in statistics means “a method of testing hypotheses about the mean of a small sample drawn from a normally distributed population when the population standard deviation is unknown”. We used this method to show how COVID-19 changed discourse linguistically. To show these changes we took the hits from Table 4 where there are frequencies from Google Search and Polish National Corpus. However, Polish National Corpus has some limitations. The database was not developed since 2010, it means that the terminology was found in texts created before 2010.

In the Tables 3 and 4, we will find the extracted terms from the previously discussed newspapers. The authors chose them as the frequency of these terms was quite high in almost every researched source. The authors divided the terms in two groups: (1) terminology strictly coined during the COVID-19 pandemic, and (2) terminology known and used before the outbreak of COVID-19 pandemic. These two groups of terms were chosen in order to check in what way COVID-19 pandemic enriched Polish language of new terminology and new concepts. It appears that terminology in most cases is new and the frequency of the usage of new terms is higher than terminology which was known and used before the pandemic. Moreover, the terms that were previously known (pre-covid terminology) where not very frequent (the Polish National Corpus hits) but now because of the pandemic are widely used in all lects.

**Table 3:** The list of terminology strictly coined during the COVID-19 pandemic with Google frequency

Terminology strictly coined during the COVID-19 pandemic	Google frequency
paszport covidowy (En. COVID passport)	723 000
Unijny Certyfikat Covid (En. The EU Digital COVID Certificate)	1 780 000
certyfikat covidowy (En. COVID certificate)	529 000
cyfrowy paszport covidowy (digital COVID passport)	68 200
unijne cyfrowe zaświadczenie COVID (En. The EU Digital COVID Certificate)	108 000
przepustka covidowa (En. covid pass)	159 000
godziny dla seniorów (En. senior citizens’ hours)	34 700 000
koronaferie (En. COVID related school break)	48 800
lockdown	386 000 000
koronakryzys (En. coronacrisis)	136 000
koronatransport (En. coronatransport)	47 000 000*
koronafobia (En. coronafobia)	2 260
koronasceptyk (En. coronasceptic)	75 900
covidiota (En. covidiot)	849 000
wariant brytyjski (En. British variant)	883 000
wariant południowoafrykański (En. South-African variant)	41 300
wariant indyjski (En. Indian variant)	603 000

*Data collected as of 29–30.09.2021*

*High frequency because of the transport company’s name KORONA

**Table 4:** The list of terminology known and used before the outbreak or COVID-19 pandemic with Google frequency and hits from Polish National Corpus (own elaboration)

Terminology known and used before the outbreak or COVID-19 pandemic	Google frequency	Polish National Corpus (data till 2010)
teleporada (En. phone doctor’s consultation)	182 000	0
cyfrowy bliźniak (En. digital twin)	274 000	0
NOP (En. VAE)	27 500 000	82
niepożądany odczyn poszczepienny (En. vaccine adverse event)	140 000	1
pandemia (En. pandemic)	434 000 000	40
epidemia (En. epidemic)	46 000 000	710
namordnik (En. muzzle)	48 700	1

*Data collected as of 29–30.09.2021*

We use *w* to denote the word that we intend to compare in two data bases (dictionaries) i.e. Google and PNC. We express the relative frequency of word *w* in database D as freq(w, D). We present Student’s t-test and check null hypothesis of a word having an equal frequency in two dictionaries, freq(w, Google) = freq(w, PNC). Alternative hypothesis is that those frequencies are different.

Denote $\alpha$ as statistical significance and let *α* = 0.05, *n*_1_, *n*_2_ number of samples where *n*_1_ = *n*_2_ = 7. Degrees of freedom is *n*_1_ + *n*_2_ − 2 = 12.

$$t = \frac{{\overline{{X_{1} }} - \overline{{X_{2} }} }}{{\sqrt {\frac{{\left( {n_{1} *s_{{X_{1} }}^{2} + n_{2} *s_{{X_{2} }}^{2} } \right)*\left( {n_{1} + n_{2} } \right)}}{{\left( {n_{1} + n_{2} - 2} \right)*n_{1} *n_{2} }}} }}$$

$$\overline{{X_{1} }} = \frac{182000 + 274000 + 27500000 + 140000 + 434000000 + 46000000 + 48700}{7}$$

$$\overline{{X_{2} }} = \frac{0 + 0 + 82 + 1 + 40 + 710 + 1}{7}$$

$s_{{X_{1} }}^{2}$ and $s_{{X_{2} }}^{2}$ are standard deviations and $s_{{X_{1} }}^{2} = 22048727042117140$ and $s_{{X_{2} }}^{2} = 59008.693878$.

At last $$t = 1,197492$$.

0.95 quantile for *df* = 12 can be read from quantiles’ table and it is equal to 1782.

*t* value belongs to non-rejection region as: $- 1782 \le t \le 1782$.

This means that null hypothesis is not rejected.

## Conclusions

After analysing the press published in the period from 1st March 2020 till 31st May 2021, some features of the narrative related to the prevailing COVID-19 pandemic can be noted, for example the use of specialist terms to describe new phenomena characteristic for the pandemic in general language. Language as a living and changing matter reacts to change and by including in the media narrative topics that affect virtually all spheres of life, these changes are reflected in the infiltration of specialist terms that are increasingly used in general language. Most of these terms have a legal basis and result mainly from legal regulations issued to establish rules of conduct during a pandemic, examples being the term “godziny dla seniorów” (En. senior citizens’ hours) or “teleporada” (En. doctor’s appointment online or by phone). Moreover, it is crucial to highlight the appearance of neologisms, which aim to describe new phenomena or objects. In Polish, these are adjectives or compound nouns with the element of COVID-19. We can also observe the appearance of borrowings, which is certainly a result of general interest in the topic and raising it also in the English-language press, e.g. NOP (En. VAE) or lockdown (terms that have passed from the medical language and have become fixed in the Polish press in an unchanged form as a borrowing). An interesting observation is also the use of colors to describe zones according to the number of infected persons. Also, the journalistic narrative on the pandemic is very dynamic and abounds in stylistic devices such as comparisons and metaphors, as described in the article. In press the three lects appear: legal lect (the terms borrowed from Polish Acts and Regulations), medical lect (COVID specific terms or medical related terms necessary to understand the “COVID language”) and journalistic lect (presented information must be understood by general public and at the same time must be “catchy”). In press there are cases of terminology which appears only in one lect (for example the term “koronaferie” or metaphors or similes which are not present in legal or medical lect). There are also cases that all three lects exist in relationship as the same term is being used in each lect, for example “Unijny Certyfikat Covid” (En. The EU Digital COVID Certificate), but in journalistic lect, also other terms are being present. Moreover, new meaning of colours appears from semiotic perspective (division into regions according to the infection rate (Kauppinen-Räisänen, Jauffret, 2018)). In Poland the colour code referring to areas where we can move and where we cannot is also inconsistent. As green and red areas, or even “zones”, the one in between has two different colours are used interchangeably—yellow and orange. As Covid-19 pandemic widespread worldwide, colours revealed not only state of alert about the virus’s threat level but also intercultural differences (for example Colorado set up a six-color system, with two shades of yellow, and a level of purple above red and Poland a four-colour system). Also during the pandemic, symbols were widespread in the press, such as the visualization of the coronavirus particle as a sphere with suction cups.

To sum up, student’s t-test shows that COVID changed the discourse linguistically. The COVID-19 pandemic affected the specialization of the journalistic discourse and introduction of the terminology from different domains into the press discourse. The increased occurrence of specific terminology from the medical and legal domain but also COVID neologisms can be noticed. Moreover, the analyzed examples indicate an increasing specialization of texts, but also an increased interdisciplinarity of texts and their intersemiotic character.

With regard to the research question of change, language is in a constant state of flux due to the magnitude of the phenomenon, and the pandemic also had a significant impact on language, particularly terminology. The subject described indicates a high dynamism of change and therefore further research into this phenomenon is advisable.

## Footnotes

1 Original version: sprachlichen Mittel[n], die in einem fachlich begrenzbaren Kommunikationsbereich verwendet werden, um die Verständigung zwischen den in diesem Bereich tätigen Menschen (und die Popularisierung der fachlichen Inhalte sowie den Kontakt zu bestimmten Nicht-Fachleuten) zu gewährleisten (Hoffmann 1984: 53).

2 Coronavirus infection is not a new disease. The earliest reports of a coronavirus infection in animals occurred in the late 1920s, when an acute respiratory infection of domesticated chickens emerged in North America. Estola T (1970). “Coronaviruses, a New Group of Animal RNA Viruses”. Avian Diseases. 14 (2): 330–336. 10.2307/1588476. ISSN 0005–2086. JSTOR 1,588,476. PMID 4,316,767.

3 “The EU vaccine ‘passport’ and what it means for travel”. BBC News. 13 July 2021. Retrieved 22 July 2021.

4 Rozporządzenie Rady Ministrów z dnia 6 maja 2021 r. w sprawie ustanowienia określonych ograniczeń, nakazów i zakazów w związku z wystąpieniem stanu epidemii.

5 CST Editorial Board (17 August 2021). “Americans need a better vaccination ID than a flimsy piece of paper”. CNN.

6 Edmond C (19 June 2020). “COVID-19: What are immunity passports and how would they work?”. World Economic Forum.

7 Smith-Spark L (3 April 2020). “Is this how to get out of lockdown?”. Chicago Sun-Times.

8 “Explained: Are immunity passports, release certificates the way to go?” indianexpress.com. 10 May 2020.

9 https://www.who.int/home/cms-decommissioning [accessed on 31.07.2021].

10 Li, Rita Yi Man; Yue, Xiao-Guang; Crabbe, M. James C. (2021). “COVID-19 in Wuhan, China: Pressing Realities and City Management”. Frontiers in Public Health. 10.3389/fpubh.2020.596913. hdl:10,547/624826. ISSN 2296–2565.

11 https://www.britannica.com/science/Students-t-test [accessed on 17.12.2021].
